# Supplementary material for: Cryo-electron tomography reveals structural insights into the membrane remodeling mode of dynamin-like EHD filaments
Source: Nat Commun. 2022 Dec 10;13:7641. doi: 10.1038/s41467-022-35164-x (PMC9741607; doi:10.1038/s41467-022-35164-x)
Supplement: Supplementary file 1 — Supplementary Information [file 41467_2022_35164_MOESM1_ESM.pdf]

Supplementary Information

Cryo-electron tomography reveals structural insights into the membrane remodeling mode of dynamin-like EHD filaments

Arthur A. Melo[1,2*], Thiemo Sprink[1,3], Jeffrey K. Noel[1], Elena Vázquez Sarandeses[1,2], Chris van Hoorn[1], Saif Mohd[1,2], Justus Loerke[4], Christian M. T. Spahn[4], Oliver Daumke[1,2*]

[1]Max-Delbrück-Center for Molecular Medicine in the Helmholtz Association, Structural Biology, Robert-Rössle-Straße 10, 13125 Berlin, Germany

[2]Freie Universität Berlin, Institute of Chemistry and Biochemistry, Takustraße 6, 14195 Berlin, Germany

[3]Cryo-Electron Microscopy Core Facility, Charité - Universitätsmedizin Berlin at the MDC, Robert-Rössle-Straße 10, 13125 Berlin, Germany

[4]Institut für Medizinische Physik und Biophysik, Charité - Universitätsmedizin Berlin, 10117 Berlin, Germany

*arthur.melo@ucsf.edu, oliver.daumke@mdc-berlin.de

Table of Contents

Supplementary Fig. 1: Structural insights into EHD function.
Supplementary Fig. 2: Structural characterization of EHD4$^{\Delta N}$-mediated membrane remodeling.
Supplementary Fig. 3: EHD2-mediated membrane remodeling.
Supplementary Fig. 4: Determination of the cryo-ET structure.
Supplementary Fig. 5: Fit of the EHD4 dimer in the cryo-ET density.
Supplementary Fig. 6: Displacement of the EH domains.
Supplementary Fig. 7: Models with alternative curvature and twist do not fit the experimental data.
Supplementary Table 1: Cryo-EM data collection, refinement and validation statistics.
Supplementary Table 2: Reported average resolutions of cryo-ET maps of vertebrate proteins obtained by STA in the Electron Microscopy Database.
Supplementary References

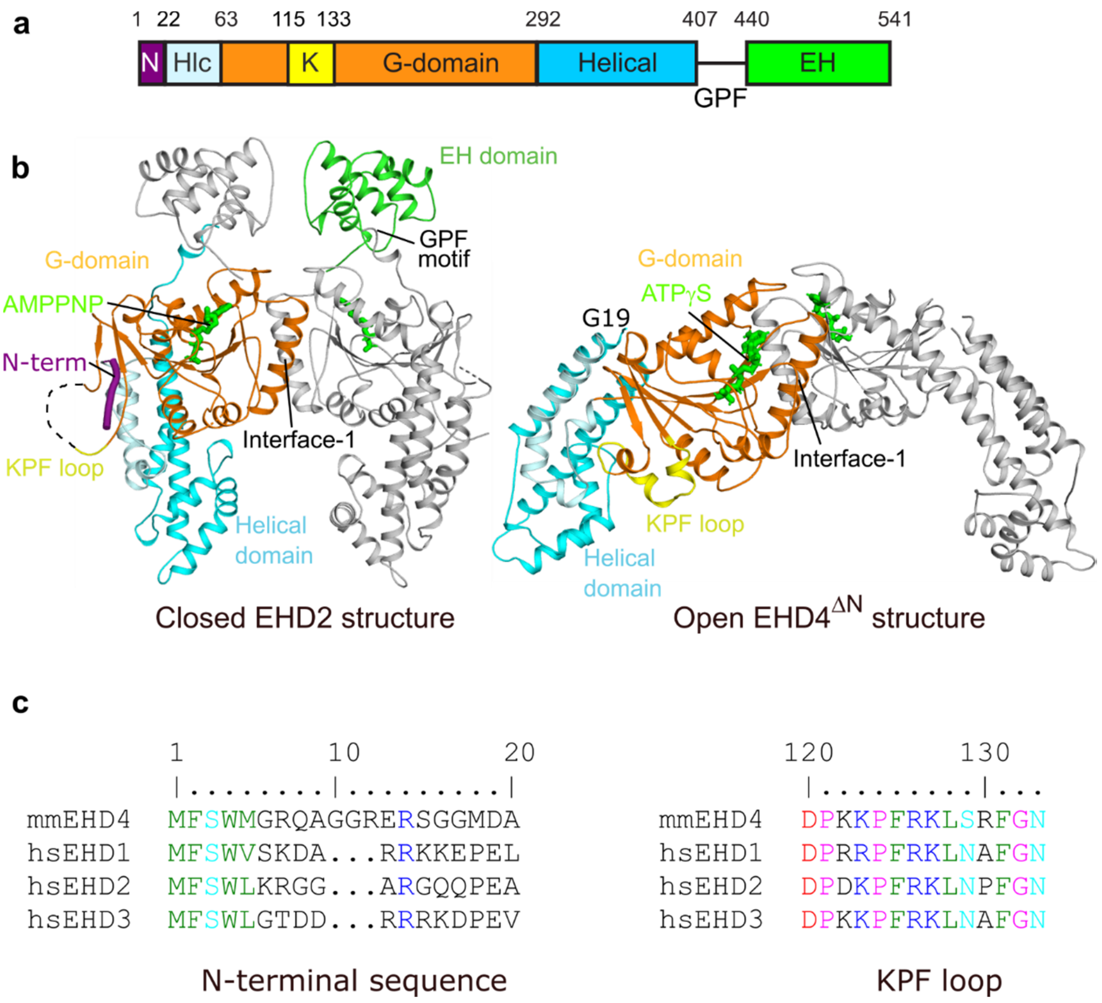

Supplementary Fig. 1. Structural insights into EHD function. a Domain architecture of EHD. N – N-terminal sequence stretch; Hlc - Helical domain; K – KPF loop; GPF-internal GPF motif; EH – EH-domain. **b** Previous structural and mechanistic insights into EHD function. In the closed, AMPPNP-bound EHD2 structure (pdb 4CID), dimerization is mediated by interface-1 in the G-domain. An N-terminal sequence stretch folds back in a hydrophobic pocket of the G-domain. The KPF loop at the distal side of the G-domain is disordered. The EH domains bind to an internal GPF motif in between the helical and EH domain on the top of the G-domain, with their C-terminal tails inserting in the nucleotide-binding site. The N-terminal sequence stretch was suggested to switch into the membrane. Right - ATPγS-bound structure of EHD4$^{\Delta N}$ (pdb 5MTV). Gly19 (G19) marks the first visible amino acid in the EHD4$^{\Delta N}$ structure. As in the closed conformation, interface-1 mediates dimerization. The KPF loop moves into the hydrophobic pocket, adopting a defined position required for assembly. The EH domains are displaced from top of the G-domain. In the open EHD4$^{\Delta N}$ crystal structure, interface-2 is formed leading to a linear oligomer in the crystal. The G-interface (interface-3) was not properly defined in any of the two structures. **c** Sequence alignments of the N-terminal sequence stretch and the KPF loop. Type-conserved residues are colored according to their physico-chemical features (green: hydrophobic, cyan: polar, blue: basic, red: acidic, magenta: proline and glycine).

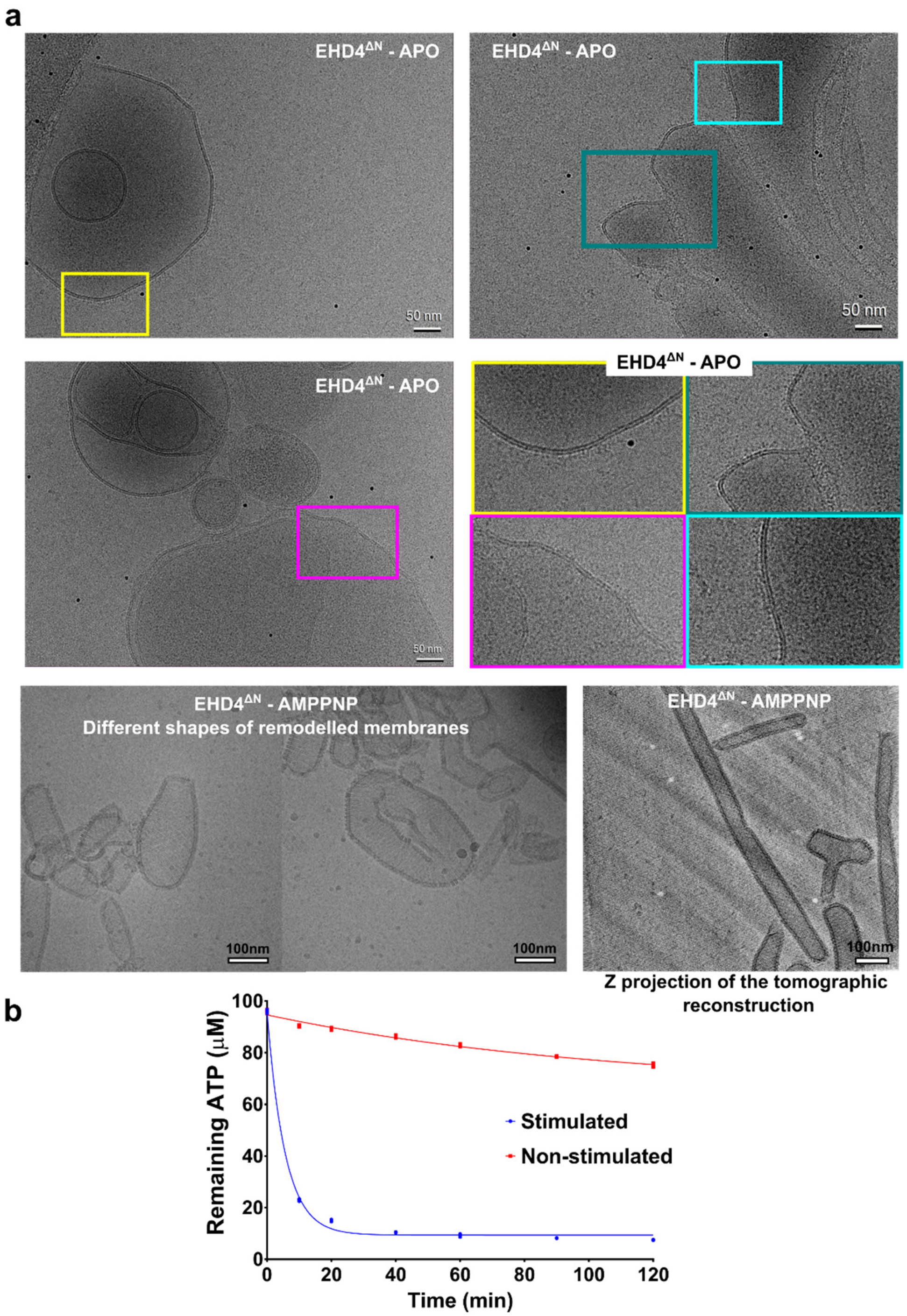

Supplementary Fig. 2. Structural characterization of EHD4$^{\Delta N}$-mediated membrane remodeling. a Representative cryo-EM micrographs of EHD4$^{\Delta N}$ in the absence and presence of AMPPNP. Insets display the indicated magnifications of the apo state, showing that nucleotide-free EHD4$^{\Delta N}$ can bind to the surface of liposomes, but does not assemble into a membrane-remodeling filament. n=2 for the apo state and n>= 10 for the AMPPNP-bound state. **b** ATPase activity of EHD4$^{\Delta N}$ at 30 °C in the presence and absence of liposomes. For each time point, three data points of technical replicates are displayed. The standard deviation is smaller than the size of each datapoint and therefore not displayed. Source data are provided as a Source Data file.

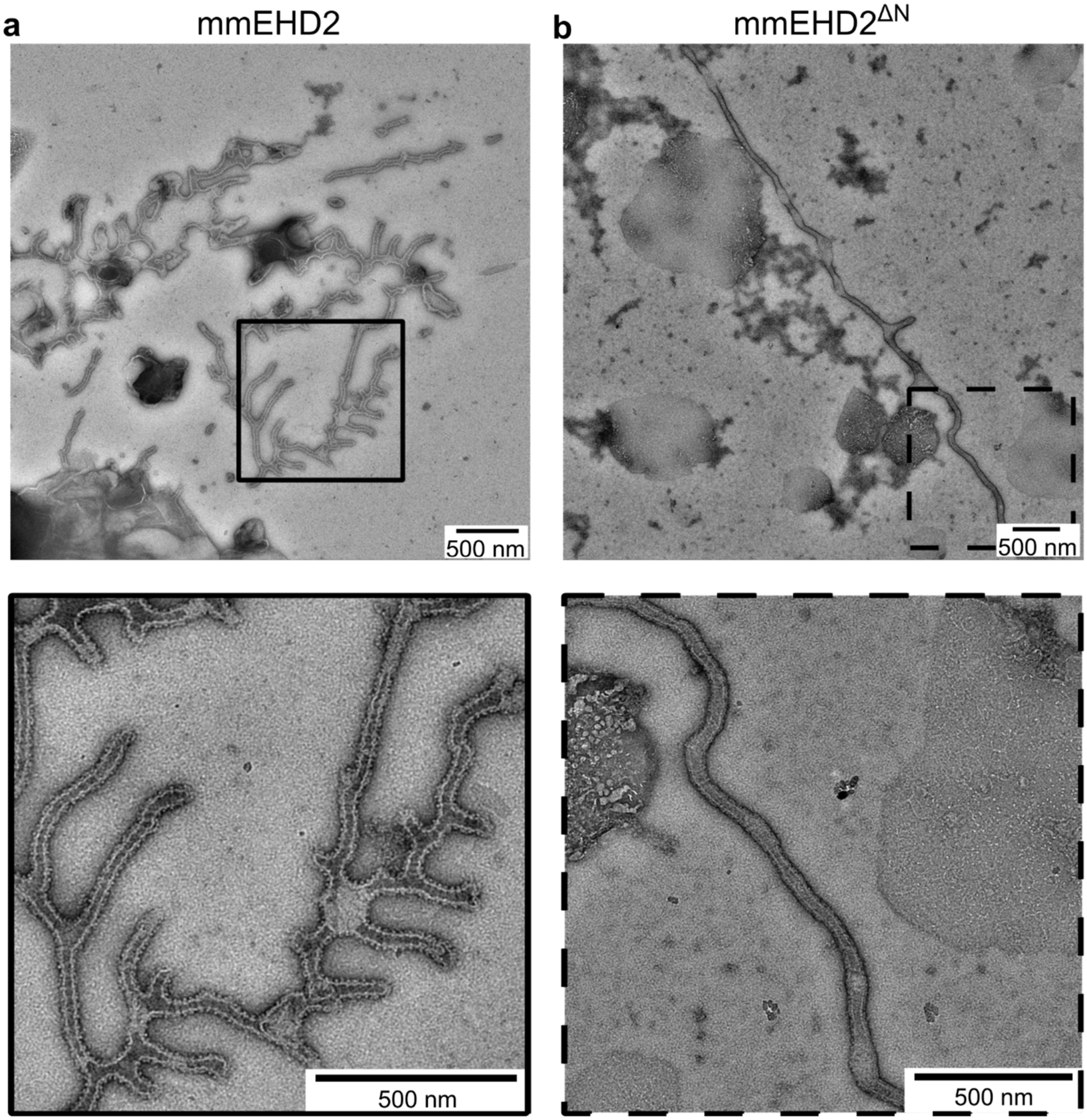

Supplementary Fig. 3. EHD2-mediated membrane remodeling. Representative negative-stain electron micrographs showing (**a**) mouse (mm)EHD2- and (**b**) mmEHD2$^{\Delta N}$-mediated liposome remodeling in the presence of ATP. EHD2 deforms liposomes to much smaller tubes than EHD4$^{\Delta N}$, and this activity is not dependent on its N-terminus. n=4.

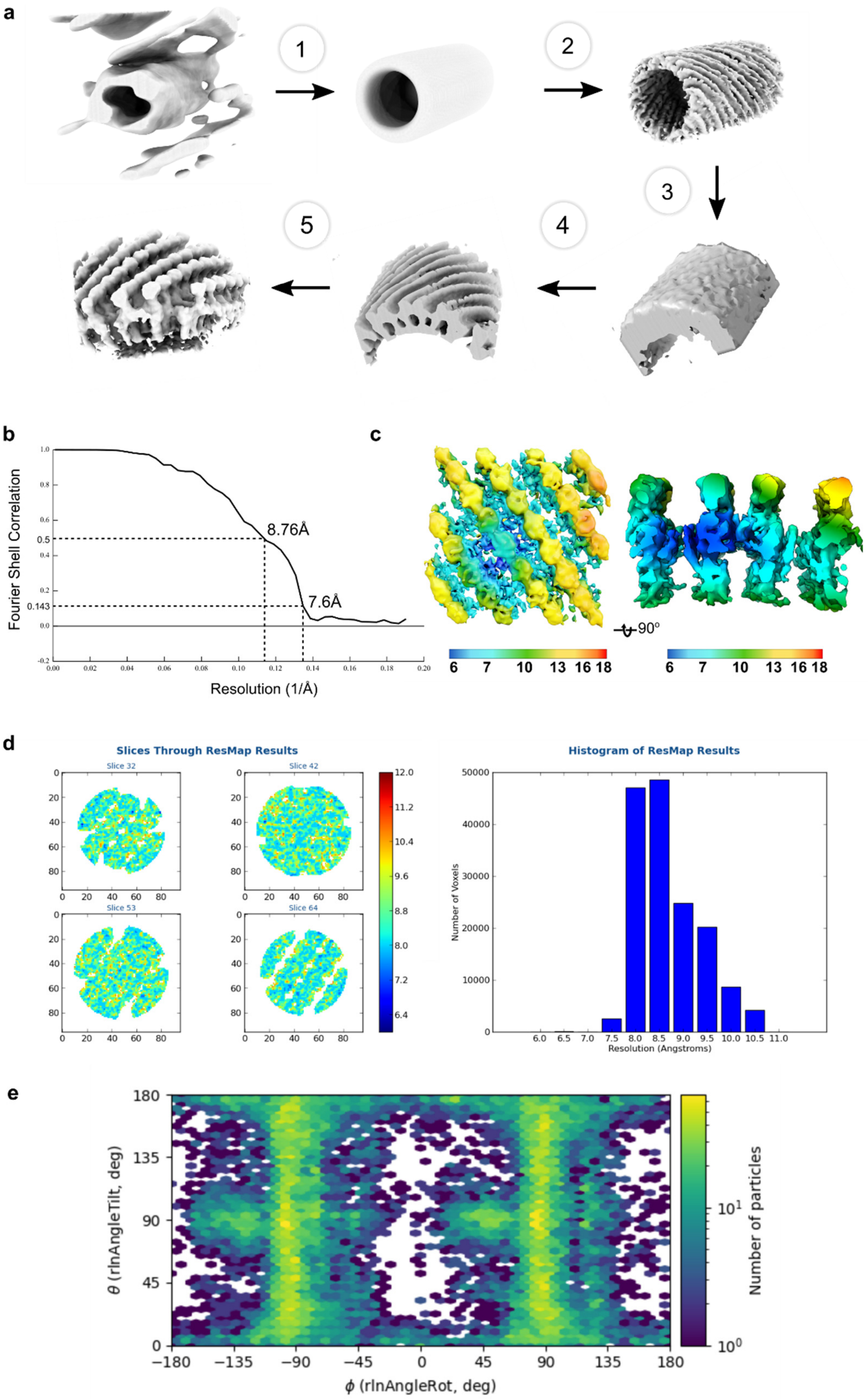
a
1
2
3
4
5
b
Fourier Shell Correlation
Resolution (1/Å)
8.76Å
7.6Å
0.5
0.143
c
90°
6 7 10 13 16 18
6 7 10 13 16 18
d
Slices Through ResMap Results
Slice 32
Slice 42
Slice 53
Slice 64
Histogram of ResMap Results
Number of Voxels
Resolution (Angstroms)
e
θ (rlnAngleTilt, deg)
ϕ (rlnAngleRot, deg)
Number of particles

Supplementary Fig. 4. Determination of the cryo-ET structure. a Subtomogram averaging workflow. Due to the large differences in tube diameter, each tube was individually cropped and averaged. A single tube shares the same missing wedge with other particles within the same tube. 1) Random rotation along the particle azimuth to generate the initial template. 2) Subtomogram averaging of individual tubes using Dynamo (see methods for more detail). This step was done to speed up subsequent processing and limit the search range for smaller particles 3) Sub-boxing along the tube walls to reduce particle size, averaging. 4) Alignment of sub-particles to the template 5) Cropping sub-particles in unbinned tomograms and subtomogram averaging with unbinned data. **b** Mask-corrected gold standard Fourier Shell Correlation. **c** Local resolution estimated with Phenix[62]. **d** Local and average resolution determination with the program ResMAP that uses FSC-independent density features to determine local resolution (Supplementary ref. 1). **e** Angular distribution of particles was calculated with the program angdist version 1.2.

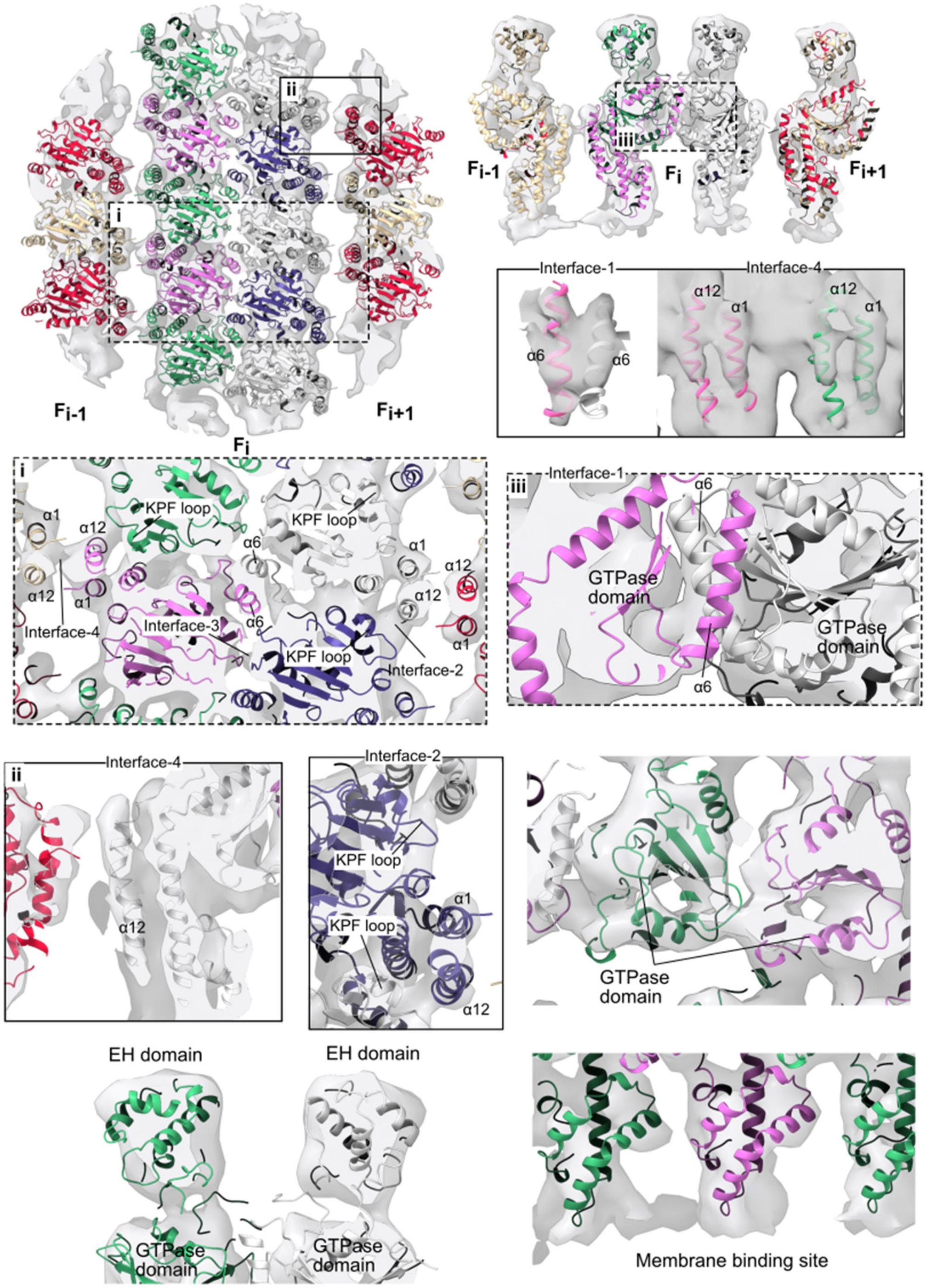

Supplementary Fig. 5. Fit of the EHD4 dimer in the cryo-ET density. A homology model of the EHD4 dimer, based on the experimentally determined closed EHD2 structure, was fitted into the determined cryo-ET density without requiring major domain shifts. The domains could therefore be orientated with high confidence into the density (Supplementary Movie 2). In agreement with the reported resolution (Supplementary Fig. 4), helical density was apparent in the most highly resolved regions of the map such as interface-1 or interface-4, further confirming our fittings.

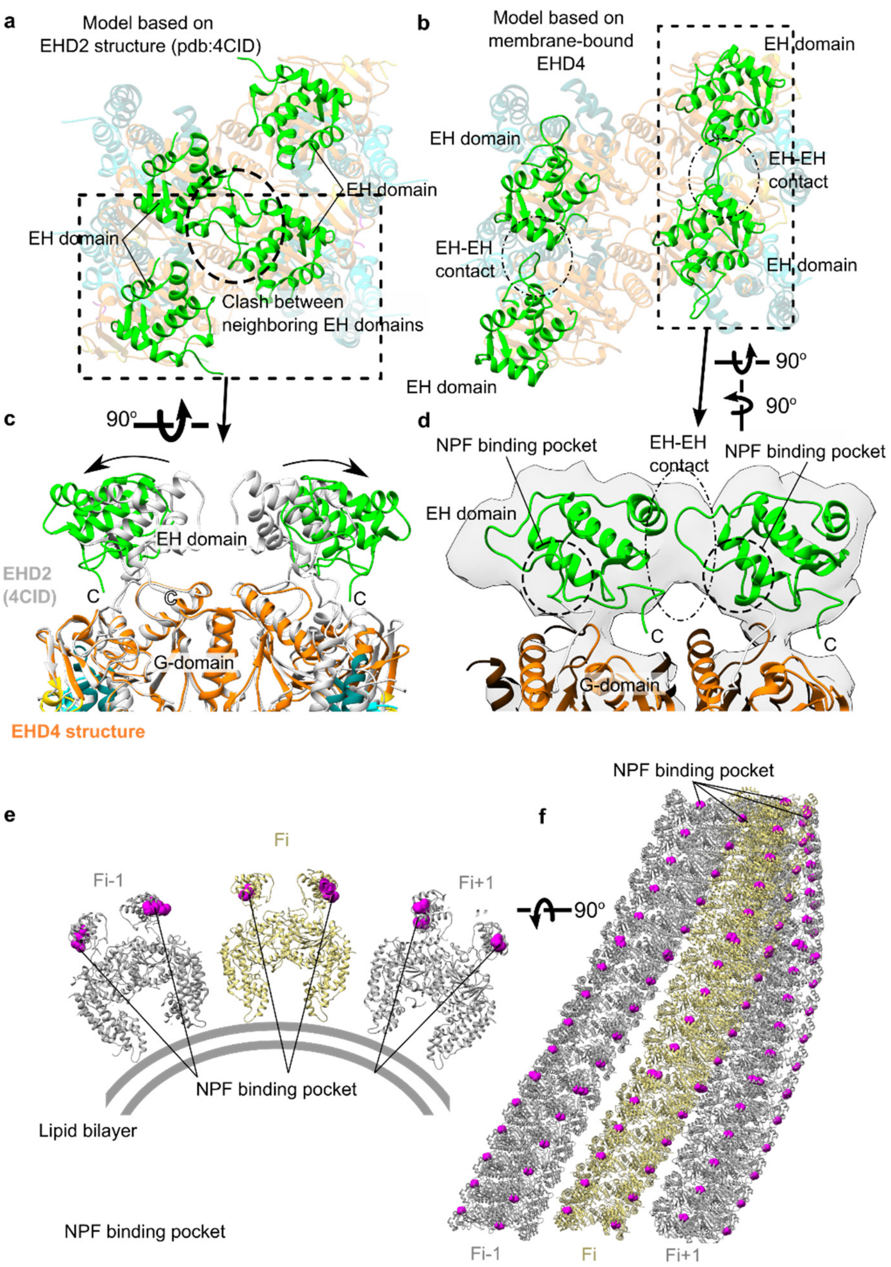

Supplementary Fig. 6. Displacement of the EH domains. a The position of the EH domain was modelled in the filament, based on the EHD2 crystal structure. In this position, EH domains of adjacent dimers would clash. **b** EH domains of adjacent dimers contact each other in the membrane-bound EHD4 structure. **c** Superposition of the membrane-bound EHD4 and EHD2 crystal structure dimer (grey). Upon membrane binding, the EH domains (green) move towards the periphery of the filament. The C-terminus of the EH domains folds into the nucleotide-binding pocket of the G-domains in the EHD2 crystal structure but is displaced in the membrane-bound structure. **d** Side view on the filament showing the new EH domain contact. Front view (**e**) and top view (**f**) of NPF binding pockets highlighted in the EHD4 filament. They are positioned on the outer region of the EHD4 filament and may be available for interactions with NPF-containing proteins in the cytosol or at the membrane.

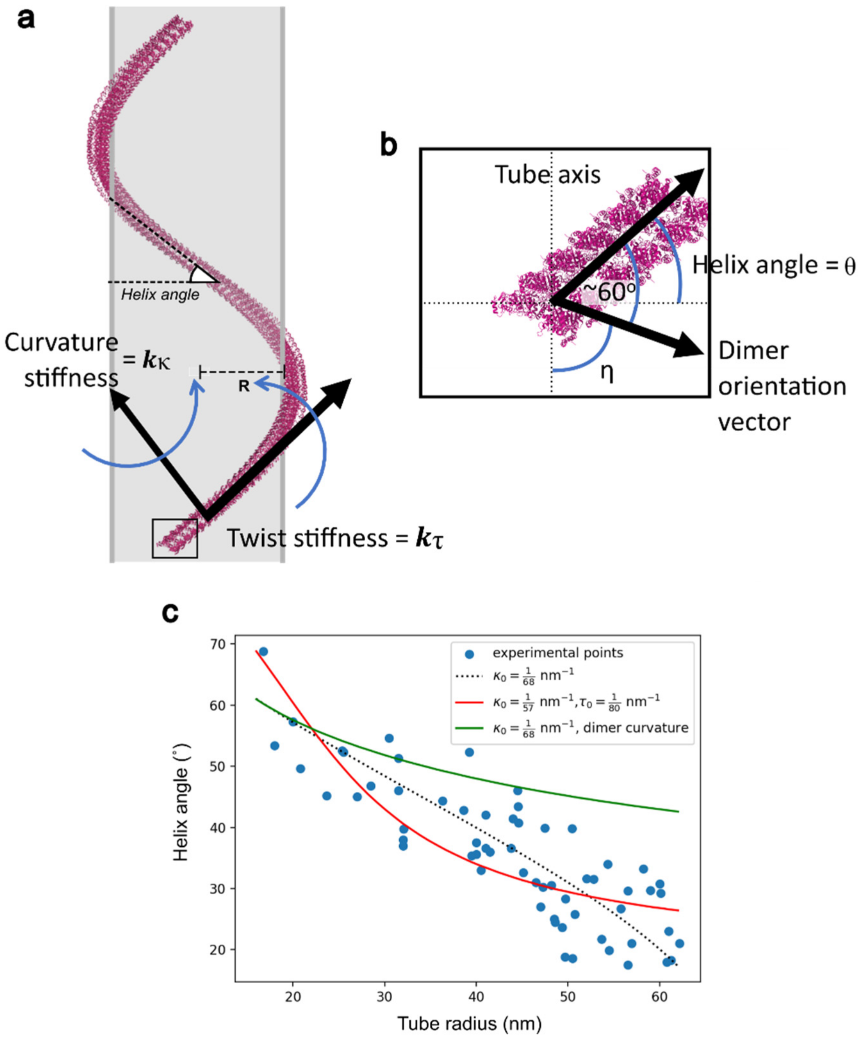

Supplementary Fig. 7. Models with alternative curvature and twist do not fit the experimental data. **a** A filament must curve around two axes in order to form a straight helix. Twist is the rotation around the vector along the helix direction with stiffness k_τ, and curvature twist is the rotation along the perpendicular vector lying in the plane of the cylinder with stiffness k_κ. **b** The filament makes contact with the membrane tube with its membrane binding sites and the dimer's intrinsic curvature is aligned with the membrane tube curvature when the dimer orientation vector η relative to the tube axis equals 90°. Since the dimer orientation with respect to the filament direction is approximately a constant 30°, η can be defined as η = θ + 30°. If the dimer curvature's alignment with the tube curvature was an important energetic component in determining the tube width, the data should align better to a model with $k_\eta > 0$. We find that $k_\eta \sim 0$ is a better fit, meaning that there is no evidence that the dimer footprint is a significant determinant of the helical curvature (see also Methods). **c** Dotted line shows best fit result of main text Fig. 5. Red line shows best fit when taking the curvature and twist moduli as equal. While the fit is not obviously wrong, the better fit of the dotted line (where twist modulus is zero) suggests the twist modulus is relatively small. The green line shows the curve given by the dotted line plus an addition accounting for an alignment driven by the dimer curvature. The large tube behavior significantly deviates. See Methods for details. Source data are provided as a Source Data file.

Supplementary Table 1: Cryo-EM data collection, refinement and validation statistics.

	**$EHD4^{\Delta N}$ (EMDB-25362) (PDB 7SOX)**
**Data collection and processing**	
Magnification	53,000
Voltage (kV)	300
Electron exposure (e–/Å^2)	94
Defocus range (μm)	3-6
Pixel size (Å)	2.628
Symmetry imposed	C2
Initial particle images (no.)	84,000
Final particle images (no.)	23,813
Map resolution (Å)	7.6
FSC threshold	0.143
Map resolution range (Å)	6.47-16
**Refinement**	
Initial model used (PDB code)	4CID
Map sharpening *B* factor (Å^2)	-250
Model composition	
Non-hydrogen atoms	66,512
Protein residues	8,224
Ligands	0
R.m.s. deviations	
Bond lengths (Å)	0.005
Bond angles (°)	0.959
Validation	
MolProbity score	2.43
Clashscore	32.98
Poor rotamers (%)	0.22
Ramachandran plot	
Favored (%)	93.48
Allowed (%)	6.52
Disallowed (%)	0.0

Supplementary Table 2: Reported average resolutions of cryo-ET maps of vertebrate proteins obtained by STA in the Electron Microscopy Database.

Resolution range	No. of reported structures
>= 2 Å and < 3 Å	1
>= 3 Å and < 4 Å	3
>= 4 Å and < 5 Å	3
>= 6 Å and < 8 Å	6
>= 8 Å and < 12 Å	32
>= 12 Å and < 16 Å	23
>= 16 Å and < 20 Å	15
>= 20 Å and < 30 Å	57
>= 30 Å	89
Total	229

Recent examples include: Pentagon bounding clathrin legs in clathrin coats assembled on a membrane. Resolution 7.7 Å. EMD-10753 (Supplementary ref. 2); an Oxysterol-binding protein (OSB) complex. Resolution 9.8 Å, EMD-11376 (Supplementary ref. 3); Endocytic complex II bound to membrane-attached Rab5a-GTP. Resolution 9.8 Å. EMD-12214 (Supplementary ref. 4); VPS26 dimer region of metazoan membrane-assembled retromer:SNX3 complex. Resolution 9.5 Å. EMD-12221 (ref. [49]); AP2 in clathrin coats assembled on a membrane containing dileucine- and tyrosine-based cargo peptides. Resolution 10.2 Å. EMD-10751 (Supplementary ref. 2). All data were extracted on April 21, 2022.

Supplementary References

1. Kucukelbir, A., Sigworth, F. J. & Tagare, H. D. Quantifying the local resolution of cryo-EM density maps. *Nat Methods* **11**, 63-65, doi:10.1038/nmeth.2727 (2014).

2. Kovtun, O., Dickson, V. K., Kelly, B. T., Owen, D. J. & Briggs, J. A. G. Architecture of the AP2/clathrin coat on the membranes of clathrin-coated vesicles. *Sci Adv* **6**, eaba8381, doi:10.1126/sciadv.aba8381 (2020).

3. de la Mora, E. *et al.* Nanoscale architecture of a VAP-A-OSBP tethering complex at membrane contact sites. *Nat Commun* **12**, 3459, doi:10.1038/s41467-021-23799-1 (2021).

4. Tremel, S. *et al.* Structural basis for VPS34 kinase activation by Rab1 and Rab5 on membranes. *Nat Commun* **12**, 1564, doi:10.1038/s41467-021-21695-2 (2021).
