# Supplementary material for: Cryo-electron tomography reveals structural insights into the membrane remodeling mode of dynamin-like EHD filaments
Source: Nat Commun. 2022 Dec 10;13:7641. doi: 10.1038/s41467-022-35164-x (PMC9741607; doi:10.1038/s41467-022-35164-x)
Supplement: Supplementary file 3 — Description of Additional Supplementary Files [file 41467_2022_35164_MOESM3_ESM.pdf]

Description of Additional Supplementary Files

File Name: Supplementary Movie 1
Description: Orthogonal views of the reconstructed tomogram containing EHD4 coated tubes.

File Name: Supplementary Movie 2
Description: Flexible fitting of the membrane-bound EHD4 filament into the cryo-ET density, starting with the closed, EHD2 crystal structure-based conformation.

File Name: Supplementary Movie 3
Description: Oligomerization of EHD4 in the filaments is mediated by three assembly interfaces.

File Name: Supplementary Movie 4
Description: Transition between the crystal structure-based linear EHD4 filament in the open conformation and the cryo-ET based, membrane-bound curved EHD4 filament in the closed conformation.
