# Supplementary material for: Cryo-electron tomography reveals structural insights into the membrane remodeling mode of dynamin-like EHD filaments
Source: Nat Commun. 2022 Dec 10;13:7641. doi: 10.1038/s41467-022-35164-x (PMC9741607; doi:10.1038/s41467-022-35164-x)
Supplement: Supplementary file 8 — Reporting Summary [file 41467_2022_35164_MOESM8_ESM.pdf]

Corresponding author(s): Melo, Daumke
Last updated by author(s): Nov 6, 2022

Reporting Summary

Nature Portfolio wishes to improve the reproducibility of the work that we publish. This form provides structure for consistency and transparency in reporting. For further information on Nature Portfolio policies, see our Editorial Policies and the Editorial Policy Checklist.

Statistics

For all statistical analyses, confirm that the following items are present in the figure legend, table legend, main text, or Methods section.

n/a	Confirmed	
☐	☒	The exact sample size (*n*) for each experimental group/condition, given as a discrete number and unit of measurement
☐	☒	A statement on whether measurements were taken from distinct samples or whether the same sample was measured repeatedly
☒	☐	The statistical test(s) used AND whether they are one- or two-sided<br>*Only common tests should be described solely by name; describe more complex techniques in the Methods section.*
☒	☐	A description of all covariates tested
☒	☐	A description of any assumptions or corrections, such as tests of normality and adjustment for multiple comparisons
☐	☒	A full description of the statistical parameters including central tendency (e.g. means) or other basic estimates (e.g. regression coefficient) AND variation (e.g. standard deviation) or associated estimates of uncertainty (e.g. confidence intervals)
☒	☐	For null hypothesis testing, the test statistic (e.g. *F*, *t*, *r*) with confidence intervals, effect sizes, degrees of freedom and *P* value noted<br>*Give P values as exact values whenever suitable.*
☒	☐	For Bayesian analysis, information on the choice of priors and Markov chain Monte Carlo settings
☒	☐	For hierarchical and complex designs, identification of the appropriate level for tests and full reporting of outcomes
☒	☐	Estimates of effect sizes (e.g. Cohen's *d*, Pearson's *r*), indicating how they were calculated

Our web collection on statistics for biologists contains articles on many of the points above.

Software and code

Policy information about availability of computer code

Data collection	Thermo Scientific - Server 2.15.3, SerialEM 4.0
Data analysis	IMOD 4.11, MotionCor2 1.1.0, CTFFIND 4.1.10, Dynamo 1.1.157, MATLAB version r2020b, RELION 3.1, Phenix 1.20, MDfit with Gromacs 4.5.5, SMOGv2.3beta, GraphPad Prism 7.05, PyMol 2.5.0, Chimera 1.14, ChimeraX 1.1, Blender 3.1, Python library scipy.optimize.least_squares 1.7.1, Visual Molecular Dynamics (VMD) 1.9.3, Ad hoc dynamo scripts at https://github.com/aamelo/dynamo_scripts. Angdist version 1.2 via https://github.com/Guillawme/angdist/blob/main/README.md

For manuscripts utilizing custom algorithms or software that are central to the research but not yet described in published literature, software must be made available to editors and reviewers. We strongly encourage code deposition in a community repository (e.g. GitHub). See the Nature Portfolio guidelines for submitting code & software for further information.

Data

Policy information about availability of data

All manuscripts must include a data availability statement. This statement should provide the following information, where applicable:

- Accession codes, unique identifiers, or web links for publicly available datasets
- A description of any restrictions on data availability
- For clinical datasets or third party data, please ensure that the statement adheres to our policy

The cryo-EM map of the EHD4 filaments has been submitted together with the atomic coordinates of the EHD4 asymmetric unit to the Electron Microscopy Data

Bank (EMDB, https://www.ebi.ac.uk/emdb/) and the pdb database (https://www.rcsb.org/) with EMDB entry EMD-25362 and PDB entry ID 7SOX, respectively. All data and material are available from the corresponding authors upon reasonable request.

Human research participants

Policy information about studies involving human research participants and Sex and Gender in Research.

Reporting on sex and gender	Not applicable.
Population characteristics	Not applicable.
Recruitment	Not applicable.
Ethics oversight	Not applicable.

Note that full information on the approval of the study protocol must also be provided in the manuscript.

Field-specific reporting

Please select the one below that is the best fit for your research. If you are not sure, read the appropriate sections before making your selection.

☒ Life sciences ☐ Behavioural & social sciences ☐ Ecological, evolutionary & environmental sciences

For a reference copy of the document with all sections, see nature.com/documents/nr-reporting-summary-flat.pdf

Life sciences study design

All studies must disclose on these points even when the disclosure is negative.

Sample size	No statistical methods were used to predetermine sample size. 56 tilt series were collected within the available data collection session for further cryo-ET processing. ATPase assays were done in three technical replicates for each conditions, which was sufficient to demonstrate stimulation of EHD4 ATPase activity by membranes. Sample size for cryo electron and negative stain micrographs are indicated in the corresponding figure legend, yielding representative images that were not further statistically evaluated.
Data exclusions	No data were excluded.
Replication	The number of replicates is indicated in the figure legends of each relevant data panel.
Randomization	Particles extracted from the cryo-electron tomography datasets were randomly divided into two half datasets for independent processing to estimate the local resolution. ATPase assays were not randomized since the data processing workflow is not subjective.
Blinding	Blinding is not relevant for cryo-ET structure determination since the results are not subjective. Investigators were not blinded during the ATPase assays, since the obtained parameters are not subjective.

Reporting for specific materials, systems and methods

We require information from authors about some types of materials, experimental systems and methods used in many studies. Here, indicate whether each material, system or method listed is relevant to your study. If you are not sure if a list item applies to your research, read the appropriate section before selecting a response.

Materials & experimental systems

n/a	Involved in the study
☒	☐ Antibodies
☒	☐ Eukaryotic cell lines
☒	☐ Palaeontology and archaeology
☒	☐ Animals and other organisms
☒	☐ Clinical data
☒	☐ Dual use research of concern

Methods

n/a	Involved in the study
☒	☐ ChIP-seq
☒	☐ Flow cytometry
☒	☐ MRI-based neuroimaging
